# Whole exome sequencing highlights variants in association with Keratoconus in Jordanian families

**DOI:** 10.1186/s12881-020-01112-z

**Published:** 2020-09-04

**Authors:** Tawfiq Froukh, Ammar Hawwari, Khalid Al Zubi

**Affiliations:** 1 Department of Biotechnology and Genetic Engineering, Philadelphia University, Jerash Road, Amman, 11118 Jordan; 2 Sight and Insight Eye Clinic, Amman, Jordan; 3 Faculty of Medicine, Mutah University, Karak, Jordan

**Keywords:** NGS, Genome, Ocular, Epithelial, Dry-eye

## Abstract

**Background:**

Keratoconus (KC) is usually bilateral, noninflammatory progressive corneal ectasia in which the cornea becomes progressively thin and conical, resulting in myopia, irregular astigmatism, and corneal scarring.

**Methods:**

Eight families characterized by consanguineous marriages and/or multiple keratoconic individuals were examined genetically. Whole exome sequencing was done as trio or quadro per family. The output of the filtration procedure, based on minor allele frequency (MAF) less than 0.01 for homozygous variants and MAF equals 0 for heterozygous variants, is 22 missense variants.

**Results:**

Based on the gene/protein function five candidate variants were highlighted in four families. Two variants were highlighted in one family within the genes *MYOF* and *STX2*, and one variant is highlighted in each of the other three families within the genes: *COL6A5*, *ZNF676* and *ZNF765*.

**Conclusion:**

This study is one of the very rare that highlights genetic variants in association with KC.

## Background

Keratoconus (KC) is sometimes bilateral, noninflammatory progressive corneal ectasia during which the cornea becomes progressively thin and conical, leading to myopia, irregular astigmatism, and corneal scarring. Patients with KC have cone shaped cornea (hence the name keratoconus, derived from the Greek word for cornea (‘kerato’) and cone shaped (‘conus’). It usually arises in the teenage years and progresses, eventually it stabilizes in the 3rd/4th decades [1]. The clinical phenotypes of KC are highly variable; however, the common feature is corneal steepening which is normally detected at an early stage of the disease using computer-assisted corneal tomography. The current treatment of keratoconus is cross linking using riboflavin (vitamin B2) and ultraviolet light, which could prevent (stop) the progression of KC. In mild cases of KC, the refractive errors are usually treated by glasses or contact lenses but in more advanced cases, surgery is required in order to restore optimal visual acuity [2].

While the prevalence of KC in Jordan is unknown it is known that the severity of KC is related to the consanguineous marriages. For example the prevalence (per 100,000) of KC is high in societies with high consanguinity such as India (2300) [3], Iran (2500) [4], Lebanon (3330) [5], and Jerusalem (2340) [6] and low in societies with low consanguinity such as UK/Caucasian (57) [7], Denmark (86) [8], Finland (30) [1], Japan (17.3) [9], and USA (54.5) [10]. In Jordan consanguineous marriage ranges between 25.5% in the capital Amman to 52.1% in Irbid (www.consang.net), and therefore KC is estimated to have comparable prevalence rates to those in Lebanon and Jerusalem [11, 12].

Aetiology of KC involves environmental factors such as eye rubbing, atopy, and allergies as well as genetic factors. Because of its heterogeneity, KC is either associated with genetic systemic disorders or exhibited in isolated cases (i.e non-syndromic; Online Mendelian Inheritance In Man, OMIM#148300) [13]. Therefore, genetic factors are expected to cause non-syndromic KC especially in consanguineous families and/or families with multiple affected individuals.

The identification of genes causing non-syndromic KC has been the focus of many studies worldwide using genome wide linkage studies and genome wide association studies. Several variants have been implicated in genes coding for the primary composition of cornea including collagens and the components of the extracellular matrix. These genes include: *LOX, CAST, DOCK9, IL1RN, SLC4A11, HGF, RAB3GAP1, TGFBI, ZNF469, ZEB1, VSX1, COL5A1, COL4A3, COL4A4, FNDC3B, FOXO1, MPDZ-NF1B, WNT10A, SOD1, IL1B, IL1A and MIR184*. However not all analyses of these genes confirm their role in KC pathogenesis [14]. Mutations in *VSX1* and *SOD1* were assigned as causative to the isolated cases of KC [15, 16]. However, contradictory results were obtained regarding the pathogenic variants within these genes. While some studies revealed missense mutations associated with KC and thus suggesting an important role of these genes in KC [15, 17–19], others found that the mutations do not segregate with the disease or do not prove the pathogenicity of the disease [15, 20]. Moreover, in the era of Next Generation Sequencing (NGS) mutations in these genes were revealed in the genome aggregation database (gnomAD; https://gnomad.broadinstitute.org [21]), suggesting that the mutations in *VSX1* and *SOD1* may not contribute to KC. Other studies have shown that mutations in *MIR184* (the most abundant expressed microRNA in the corneal and lens epithelia) are candidates (but not consistent) causing KC and cataract [22, 23]. In Jordan, NGS was recently used to identify variants causing neurodevelopment diseases [24–31]. In one study homozygous frameshift variant in the gene *GALNT14* is identified to be in association with KC [32].

In this study, Whole Exome Sequencing (WES) is used to identify rare variants associated with KC in eight Jordanian families.

## Methods

### Families recruitment

Patients with KC were recruited in July 2017 from Sight and Insight clinic/Amman in collaboration with Dr. Ammar Hiwari and from Al Karak hospital in collaboration with Dr. Khalid Al Zubi. KC was diagnosed during a routine eye test using slit lamp examination, snellen chart vision test, refraction Corneal topography by a pentacam machine “Oculus, Germany”. KC was confirmed using Belin / Ambrósio Enhanced Ectasia Display. Because the goal of this study is the identification of genetic factors being in association with KC, the targeted study group in this study is consanguineous families and/or multiple affected individuals in the same family.

Written informed consents were obtained from all participants in this study. Written informed consents were obtained from the parents of all participants under the age of 16. All participants have provided written informed consents to publish all identifying images, personal details and clinical details anonymously. Peripheral blood samples were collected from all participants.

### Molecular genetic analysis

Total genomic DNA was extracted with FlexiGene DNA kit. WES was conducted to 28 individuals (17 keratoconic and 11 healthy). Exome sequencing, bioinformatics analyses and variants validation by sanger sequencing were done as described previously [32].

### Variants filtration procedure

We identified high-quality variants that are located in the protein coding genes including variants within the two base pair flanking the splicing sites (according to Ensembl-GRCh37.73). We maintained only the variants meeting the following quality criteria: at least 20X coverage and a mapping quality score ≥ 60. Variants meetings quality criteria were grouped into homozygous (recessive model) and heterozygous (dominant model) variants. In house controls of 200 Jordanian exomes were used in the filtration procedure.

**Fig. 1:**
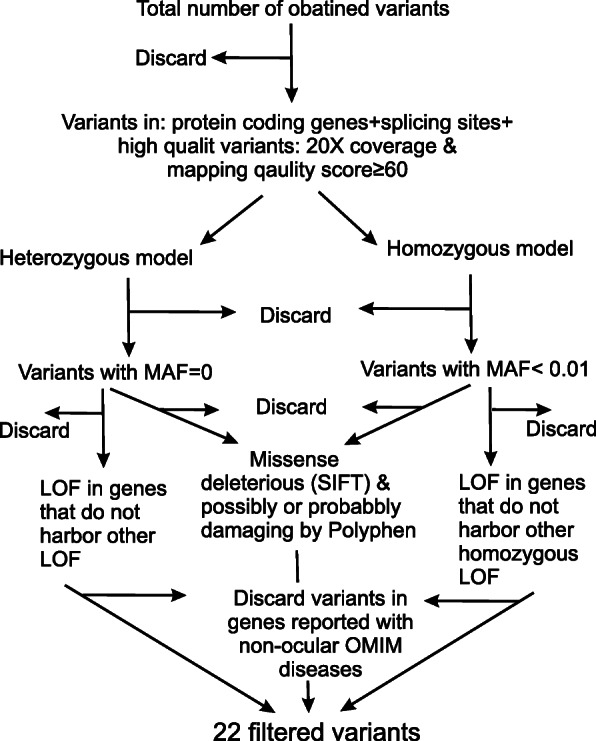
Variants filtration chart. MAF: minor allele frequency, LOF: loss of function

Homozygous variants were filtered according to the following. Rare variants with minor allele frequency (MAF) less than 0.01 in the following databases: genome aggregation database (gnomAD), 1000 genome project and in the in-house sequenced controls. Shared homozygous variants between the keratconic individuals per family but heterozygous in the parents (if not keratoconic) were maintained. Filtration was then done only for the loss of function variants (LOF; including stop gain, frameshift, splice site acceptor and splice site donor) and the missense variants predicted to be possibly- or probably-damaging by Polyphen2 Humvar and predicted to be deleterious by SIFT [33–35]. Loss of function (LOF) variants were also excluded if the genes they are located in carry other homozygous LOF variants in gnomAD, 1000 genome project or in the in-house sequenced controls. Variants located in genes reported with non-ocular OMIM disease were excluded because we aim to identify rare variants in association with non-syndromic KC (Fig. 1).

Heterozygous variants were filtered according to three criteria. Firstly, variants that are absent in the following databases: gnomAD, 1000 genome project and in the in-house sequenced controls. Secondly, shared heterozygous variants between the keratconic individuals per family but wild type in the parents (if not affected). Filtration was then done only for the loss of function variants (LOF; including stop gain, frameshift, splice site acceptor and splice site donor) and missense variants predicted to be possibly- or probably-damaging by Polyphen2 Humvar and predicted to be deleterious by SIFT. LOF variants were also excluded if the gene they are located in carry other LOF variants in gnomAD, 1000 genome project or in the in-house sequenced controls. Thirdly, variants were excluded if they are in genes reported with non-ocular OMIM disease because we aim to identify rare variants with non-syndromic KC (Fig. 1).

## Results

### Identified variants

Based on the filtration procedure, 22 variants were identified. All of the identified variants are missense. Four variants are homozygous in four families: KC003, KC004, KC010 and KC011 and 18 variants are heterozygous in five families: KC001(two variants), KC003 (three variants) KC005 (five variants), KC007 (one variants), KC008 (seven variants) (Table 1). None of the filtered variants is in a gene reported with OMIM disease.

**Table 1:** Identified variants after the filtration procedure

Family	Genotype	gene	OMIM No.	Variant
KC001	het	CDC42BPA	603,412	ENST00000334218:c.4586G > A:p.Arg1529Gln
		LRRC16B	614,716	ENST00000342740:c.2290G > A:p.Asp764Asn
KC003	hom	POLR2M	606,485	ENST00000299638:c.42G > C:p.Glu14Asp
	het	GHITM	–	ENST00000372134:c.770 T > G:p.Val257Gly
		MYOF	604,603	ENST00000359263:c.2906G > C:p.Cys969Ser
		STX2	132,350	ENST00000392373:c.636 T > G:p.His212Gln
KC004	hom	MLLT4	159,559	ENST00000400822:c.5381G > A:p.Gly1794Glu
KC005	het	GOLGA4	602,509	ENST00000356847:c.779G > A:p.Gly260Asp
		RPAP1	611,475	ENST00000304330:c.190G > A:p.Asp64Asn
		NTN3	602,349	ENST00000293973:c.142G > T:p.Ala48Ser
		TDRD12	–	ENST00000444215:c.1849 T > C:p.Trp617Arg
		SPATA25	–	ENST00000372519:c.674C > G:p.Ser225Cys
KC007	het	ZNF676	–	ENST00000397121:c.1240A > G:p.Ile414Val
KC008	het	XIRP1	609,777	ENST00000340369:c.4G > A:p.Ala2Thr
		COL6A5	611,916	ENST00000265379:c.5014 T > G:p.Phe1672Val
		HLTF	603,257	ENST00000310053:c.2269G > T:p.Asp757Tyr
		UTRN	128,240	ENST00000367545:c.9911C > A:p.Ser3304Tyr
		TBATA	612,640	ENST00000299290:c.130C > T:p.Pro44Ser
		KRTAP17–1	–	ENST00000334202:c.13C > G:p.Pro5Ala
		SYCP2	604,105	ENST00000357552:c.1278A > T:p.Gln426His
KC010	hom	NPIPB5	–	ENST00000424340:c.1353G > T:p.Lys451Asn
KC011	hom	ZNF765	–	ENST00000396408:c.1133C > T:p.Thr378Ile

## Discussion

### Family KC001

**Fig. 2:**
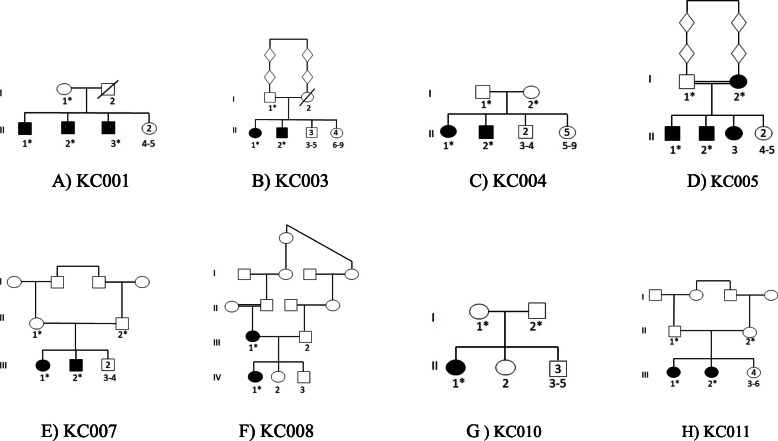
Pedigrees of the recruited families. **a** Family KC001, **b** Family KC003, **c** Family KC004, **d** Family KC005, **e** Family KC007, **f** Family KC00 8, **g** Family KC010, **h** Family KC011 * Individuals included in the study

The family has 3 keratoconic siblings born to non-consanguineous parents (Fig. 2a). WES was performed to the 3 siblings (II-1, II-2 and II-3) and to their mother (I-1). Two heterozygous missense variants out of 118,763 variants were identified in the genes *CDC42BPA* and *LRRC16B*. While no information about the gene *LRRC16B*, the gene *CDC42BPA* encodes a kinase protein that phosphorylates histone H1. This protein plays a role in the regulation of cytoskeleton and cell migration and abundant in the heart, brain, skeletal muscle, kidney and pancreas [36]. Despite the fact that the identified variants (c.4586G > A:p.Arg1529Gln in the gene *CDC42BPA* and c.2290G > A:p.Asp764Asn *LRRC16B*) in these two genes are predicted to be deleterious no relationship can be established between these two variants and KC because there are no reports link any of these two genes with ocular functions.

### KC003

Distantly related parents in this family have two keratoconic progeny (Fig. 2b). Performing WES to II-1, II-2 and I-1 yields 113,885 variants. According to the filtration procedure four variants are identified. One variant is homozygous in the gene *POLR2M* and three are heterozygous in the genes *GHITM*, *MYOF* and *STX2*.

The variants in the genes *POLR2M* and *GHITM* are excluded to be in association with keratoconus because the first encodes a subunit of a specific form of RNA polymerase II that act as negative regulator of transcriptional activation by the mediator complex [37], and the second encodes a growth hormone inducible transmembrane protein that is required for the mitochondrial tubular network and cristae organization [38]. Usually abnormalities in such functions are in association with developmental disorders.

The variants in the genes *MYOF* and *STX2* are more likely to be in association with keratoconus because *MYOF* is calcium/phospholipid-binding protein that plays a role in the plasmalemma repair mechanism of endothelial cells that permits rapid resealing of membranes disrupted by mechanical stress [39]. While the gene *STX2* encodes a protein that regulates epithelial mesenchymal interactions and epithelial cell morphogenesis and activation [40].

### KC004

The parents in this family I-1 and I-2 belong to two different families but from the same town nonetheless they have two keratoconic progeny II-1 and II-2 (Fig. 2c). WES revealed 130,125 variants and the filtration identified one variant (c.5381G > A:p.Gly1794Glu) in the gene *MLLT4* which is not likely contributing to keratoconus. This is because this gene encodes a protein involved in signalling and organization of cell junctions during embryogenesis [41]. Dysfunction here is most likely in association with central nervous system diseases and therefore, no variant can be assigned to this family in association with keratoconus.

### KC005

Distantly related parents with four keratoconic individuals including the mother were recruited in this family (Fig. 2d). The whole exome sequences in this family revealed 124,431 variants and the filtration procedure identified five variants in five different genes (Table 1). Screening the functions of the gene products of these five genes excludes any of them to be in association with keratoconus and therefore no variant can be assigned to this family.

### KC007 and KC011

While the parents in these two families are first cousin with two keratoconic progeny (Fig. 2e and h), no homozygous variants were identified but rather a heterozygous variant (c.1240A > G:p.Ile414Val) in the gene *ZNF676* for family KC007 and the variant c.1133C > T:p.Thr378Ile in the gene *ZNF765*. No detailed information is available about the proteins of these two genes except being zinc finger proteins that might be involved in transcriptional regulations. However, the identified variants in these two zinc finger proteins might be in association with KC because variants in another zinc finger protein (*ZNF469*) were previously reported in association with keratoconus [42–45].

### KC008

In this family the mother and her daughter are keratoconic (Fig. 2f). Whole exome sequence revealed 103,750 variants and the filtered are seven variants in seven genes. By searching the functions of the proteins encoded by these seven genes, only one is prioritized *COL6A5*. This gene encodes a member of the collagen VI that acts as a cell-binding protein [46].

### KC010

This is the only family in this study with one keratoconic individual who belong to nonconsanguineous parents (Fig. 2g). According to the filtration procedure only one variant was identified in the gene *NPIPB5*. This gene encodes a nuclear pore complex interacting protein which does not seem to be involved with keratoconus and therefore no variants are assigned to this family.

## Conclusion

In summary, WES and the validation by sanger sequencing allowed us to highlight variants in association with KC in four families (two variants in KC003 and one variant in each of KC007, KC008, and KC011) and no variants to highlight in three families (KC001, KC004, KC005 and KC010). The families were chosen in this study with multiple keratoconic individual as a sort of evidence for the genetic contribution on KC. Nonetheless, none of the prioritized variants in the families (KC003, KC007, KC008, and KC011) is strong candidate to cause KC. This is due to the lack of concrete information connecting between the functions of the gene products (proteins) and the cornea. Therefore, further experimental assays are needed to strengthen the possible causality of the identified variants and the candidate genes on KC.

## Data Availability

The highlighted variants in association with keratoconus in this article are available in Leiden Open Variation Database LOVD 3.0 (https://databases.lovd.nl/) according to reference genome GRCh37.73 used in this study. KC003 (*MYOF*) : https://databases.lovd.nl/shared/individuals/00307910 KC003 (*STX2*) :https://databases.lovd.nl/shared/individuals/00307911 KC007 (*ZNF676*) :https://databases.lovd.nl/shared/individuals/00307912 KC008 (*COL6A5*) :https://databases.lovd.nl/shared/individuals/00307914 KC011 (*ZNF765*) :https://databases.lovd.nl/shared/individuals/00307913

## Abbreviations

KC: Keratoconus
MAF: Minor Allele Frequency
NGS: Next Generation Sequencing
WES: Whole Exome Sequencing
OMIM: Online Mendelian Inheritance In Man
gnomAD: Genome Aggregation Database
LOF: loss of function
GRCh37.73: Genome Reference Consortium Build 37.73

## References

[1] Ihalainen A (1986). Clinical and epidemiological features of keratoconus genetic and external factors in the pathogenesis of the disease. Acta Ophthalmologica Supplement.

[2] Li X, Rabinowitz YS, Rasheed K, Yang H (2004). Longitudinal study of the normal eyes in unilateral keratoconus patients. Ophthalmology..

[3] Jonas JB, Nangia V, Matin A, Kulkarni M, Bhojwani K (2009). Prevalence and associations of keratoconus in rural Maharashtra in Central India: the Central India eye and medical study. Am J Ophthalmol.

[4] Ziaei H, Jafarinasab MR, Javadi MA, Karimian F, Poorsalman H, Mahdavi M (2012). Epidemiology of keratoconus in an Iranian population. Cornea..

[5] Waked N, Fayad AM, Fadlallah A, El Rami H (2012). Keratoconus screening in a Lebanese students' population. J Francais d’Ophtalmologie.

[6] Millodot M, Shneor E, Albou S, Atlani E, Gordon-Shaag A (2011). Prevalence and associated factors of keratoconus in Jerusalem: a cross-sectional study. Ophthalmic Epidemiol.

[7] Pearson AR, Soneji B, Sarvananthan N, Sandford-Smith JH (2000). Does ethnic origin influence the incidence or severity of keratoconus?. Eye (Lond).

[8] Bak-Nielsen S, Ramlau-Hansen CH, Ivarsen A, Plana-Ripoll O, Hjortdal J. Incidence and prevalence of keratoconus in Denmark - an update. Acta Ophthalmol. 2019;97:752–5. 10.1111/aos.14082 30964230

[9] Tanabe U, Fujiki K, Ogawa A, Ueda S, Kanai A (1985). Prevalence of keratoconus patients in Japan. Nippon Ganka Gakkai Zasshi.

[10] Kennedy RH, Bourne WM, Dyer JA (1986). A 48-year clinical and epidemiologic study of keratoconus. Am J Ophthalmol.

[11] Abu Ameerh MA, Bussieres N, Hamad GI, Al Bdour MD (2014). Topographic characteristics of keratoconus among a sample of Jordanian patients. Int J Ophthalmol.

[12] Hanan A. Hamamy ATM, Azmy M. Al-Hadidy, Kamel M. Ajlouni. Consanguinity and genetic disorders. Profile from Jordan.pdf. Saudi Med J 2007. 2007;28(7):185–92. 17603701

[13] Joel Sugar M, Marian S, Macasi M (2012). What causes Keratoconus?. Cornea..

[14] Bykhovskaya Y, Margines B, Rabinowitz YS (2016). Genetics in Keratoconus: where are we?. Eye Vis (Lond).

[15] Dash DP, George S, O'Prey D, Burns D, Nabili S, Donnelly U (2010). Mutational screening of VSX1 in keratoconus patients from the European population. Eye (Lond)..

[16] Udar N, Atilano SR, Small K, Nesburn AB, Kenney MC (2009). SOD1 haplotypes in familial keratoconus. Cornea..

[17] Bisceglia L, Ciaschetti M, De Bonis P, Campo PA, Pizzicoli C, Scala C (2005). VSX1 mutational analysis in a series of Italian patients affected by keratoconus: detection of a novel mutation. Invest Ophthalmol Vis Sci.

[18] Tang YG, Picornell Y, Su X, Li X, Yang H, Rabinowitz YS (2008). Three VSX1 gene mutations, L159M, R166W, and H244R, are not associated with keratoconus. Cornea..

[19] De Bonis P, Laborante A, Pizzicoli C, Stallone R, Barbano R, Longo C (2011). Mutational screening of VSX1, SPARC, SOD1, LOX, and TIMP3 in keratoconus. Mol Vis.

[20] Štabuc-Šilih M, Stražišar M, Ravnik Glavač M, Hawlina A, Glavač D. Genetics and clinical characteristics of keratoconus. Acta Dermatoven. 2010;19(2):3–10. 20664914

[21] Karczewski KJ, Francioli LC, Tiao G, Cummings BB, Alföldi J, Wang Q, et al. The mutational constraint spectrum quantified from variation in 141,456 humans. bioRxiv. 2020;581:434–43. 10.1038/s41586-020-2308-7 PMC7334197 32461654

[22] Hughes AE, Bradley DT, Campbell M, Lechner J, Dash DP, Simpson DA (2011). Mutation altering the miR-184 seed region causes familial keratoconus with cataract. Am J Hum Genet.

[23] Iliff BW, Riazuddin SA, Gottsch JD (2012). A single-base substitution in the seed region of miR-184 causes EDICT syndrome. Invest Ophthalmol Vis Sci.

[24] Froukh T (2019). First record mutations in the genes ASPA and ARSA causing Leukodystrophy in Jordan. Biomed Res Int.

[25] Froukh T (2019). Genetic study in a family with dopa-responsive dystonia revealed a novel mutation in sepiapterin reductase gene. Pak J Med Sci.

[26] Froukh T, Nafie O, Al Hait SAS, Laugwitz L, Sommerfeld J, Sturm M (2020). Genetic basis of neurodevelopmental disorders in 103 Jordanian families. Clin Genet.

[27] Froukh TJ (2017). Next generation sequencing and genome-wide genotyping identify the genetic causes of intellectual Disbailiuty in ten consanguineous families from Jordan. Tohoku J Exp Med.

[28] Han C, Alkhater R, Froukh T, Minassian AG, Galati M, Liu RH (2016). Epileptic encephalopathy caused by mutations in the guanine nucleotide exchange factor DENND5A. Am J Hum Genet.

[29] Johansen A, Rosti RO, Musaev D, Sticca E, Harripaul R, Zaki M (2016). Mutations in MBOAT7, encoding Lysophosphatidylinositol Acyltransferase I, Lead to intellectual disability accompanied by epilepsy and autistic features. Am J Hum Genet.

[30] Nafi ORB, Riess O, Buchert R, Froukh T (2019). Two cases of variant late infantile ceroid lipofuscinosis in Jordan. World J Clin Cases.

[31] Reuter MS, Tawamie H, Buchert R, Hosny Gebril O, Froukh T, Thiel C (2017). Diagnostic yield and novel candidate genes by exome sequencing in 152 consanguineous families with neurodevelopmental disorders. JAMA Psychiatry.

[32] Froukh T, Hawwari A (2019). Autosomal recessive non-syndromic Keratoconus: homozygous Frameshift variant in the candidate novel gene GALNT14. Curr Mol Med.

[33] Adzhubei I, Jordan DM, Sunyaev SR. Predicting functional effect of human missense mutations using PolyPhen-2. Curr Protoc Hum Genet. 2013;Chapter 7:Unit7 20. 10.1002/0471142905.hg0720s76 PMC4480630 23315928

[34] Ng PC (2003). SIFT: predicting amino acid changes that affect protein function. Nucleic Acids Res.

[35] Vaser R, Adusumalli S, Leng SN, Sikic M, Ng PC. SIFT missense predictions for genomes. Nat Protoc 2016;11(1):1–9. 10.1038/nprot.2015.123 26633127

[36] Zhao Y, Loyer P, Li H, Valentine V, Kidd V, Kraft AS (1997). Cloning and chromosomal location of a novel member of the myotonic dystrophy family of protein kinases. J Biol Chem.

[37] Hu X, Malik S, Negroiu CC, Hubbard K, Velalar CN, Hampton B (2006). A mediator-responsive form of metazoan RNA polymerase II. Proc Natl Acad Sci U S A.

[38] Oka T, Sayano T, Tamai S, Yokota S, Kato H, Fujii G (2008). Identification of a novel protein MICS1 that is involved in maintenance of mitochondrial morphology and apoptotic release of cytochrome c. Mol Biol Cell.

[39] Britton S, Freeman T, Vafiadaki E, Keers S, Harrison R, Bushby K (2000). The third human FER-1-like protein is highly similar to dysferlin. Genomics..

[40] Low SH, Li X, Miura M, Kudo N, Quinones B, Weimbs T (2003). Syntaxin 2 and endobrevin are required for the terminal step of cytokinesis in mammalian cells. Dev Cell.

[41] Taya S, Yamamoto T, Kano K, Kawano Y, Iwamatsu A, Tsuchiya T (1998). The Ras target AF-6 is a substrate of the fam deubiquitinating enzyme. J Cell Biol.

[42] Loukovitis E, Tsotridou E, Vakalis T, Asteriadis S, Sousouras T, Zachariadis Z (2019). Contributions of the superoxide dismutase 1 and zinc finger protein 469 (ZNF469) genes to keratoconus. J Biol Regul Homeost Agents.

[43] Yildiz E, Bardak H, Gunay M, Bardak Y, Imamoglu S, Ozbas H (2017). Novel zinc finger protein gene 469 (ZNF469) variants in advanced Keratoconus. Curr Eye Res.

[44] Yu X, Chen B, Zhang X, Shentu X (2017). Identification of seven novel ZNF469 mutations in keratoconus patients in a Han Chinese population. Mol Vis.

[45] Vincent AL, Jordan CA, Cadzow MJ, Merriman TR, McGhee CN (2014). Mutations in the zinc finger protein gene, ZNF469, contribute to the pathogenesis of keratoconus. Invest Ophthalmol Vis Sci.

[46] Gara SK, Grumati P, Urciuolo A, Bonaldo P, Kobbe B, Koch M (2008). Three novel collagen VI chains with high homology to the alpha3 chain. J Biol Chem.

